# Clinical impact of “pure” empirical catheter ablation of slow-pathway in patients with non-ECG documented clinical on–off tachycardia

**DOI:** 10.1186/s40001-018-0314-0

**Published:** 2018-03-27

**Authors:** Shqipe Gerguri, Nikesh Jathanna, Tina Lin, Patrick Müller, Lukas Clasen, Jan Schmidt, Muhammed Kurt, Dong-In Shin, Christian Blockhaus, Malte Kelm, Alexander Fürnkranz, Hisaki Makimoto

**Affiliations:** 1 Division of Cardiology, Pulmonology and Vascular Medicine, Medical Faculty, University Duesseldorf, Moorenstr. 5, 40225 Duesseldorf, Germany; 2 Medical Faculty, Cardiovascular Research Institute Duesseldorf (CARID), University Duesseldorf, Duesseldorf, Germany; 3 Heart Care Victoria, Victoria, Australia

**Keywords:** AVNRT, Supraventricular tachycardia, Slow-pathway ablation, Empiric ablation, ECG documentation

## Abstract

**Background:**

Catheter ablation of slow-pathway (CaSP) has been reported to be effective in patients with dual atrioventricular nodal conduction properties (dcp-AVN) and clinical ECG documentation but without the induction of tachycardia during electrophysiological studies (EPS). However, it is unknown whether CaSP is beneficial in the absence of pre-procedural ECG documentation and without the induction of tachycardia during EPS. The aim of this study was to evaluate long-term results after a “pure” empirical CaSP (peCaSP).

**Methods:**

334 consecutive patients who underwent CaSP (91 male, 47.5 ± 17.6 years) were included in this study. Sixty-three patients (19%) who had no pre-procedural ECG documentation, and demonstrated dcp-AVN with a maximum of one echo-beat were assigned to the peCaSP group. The remaining 271 patients (81%) were assigned to the standard CaSP group (stCaSP). Clinical outcomes of the two groups were compared, based on ECG documented recurrence or absence of tachycardia and patients’ recorded symptoms.

**Results:**

CaSP was performed in all patients without any major complications including atrioventricular block. During follow-up (909 ± 435 days), 258 patients (77%) reported complete cessation of clinical symptoms. There was no statistically significant difference in the incidence of AVNRT recurrence between the peCaSP and stCaSP groups (1/63 [1.6%] vs 3/271 [1.1%], *P* = 0.75). Complete cessation of clinical symptoms was noted significantly less frequently in patients after peCaSP (39/63 [62%] vs 219/271 [81%], *P* = 0.0013). The incidence of non-AVNRT atrial tachyarrhythmias (AT) was significantly higher in patients after peCaSP (5/63 [7.9%] vs 1/271 [0.4%], *P* = 0.0011).

**Conclusion:**

A higher incidence of other AT and subjective symptom persistence are demonstrated after peCaSP, while peCaSP improves clinical symptoms in 60% of patients with non-documented on–off tachycardia.

## Background

Electrophysiological study (EPS) and ablation with elimination or modulation of the slow-pathway (SP) is an established treatment for patients with atrioventricular-nodal re-entrant tachycardia (AVNRT). A high success rate with a low recurrence and complication rate has been reported [1]. The most important but rare complication is iatrogenic third degree atrioventricular (AV) block necessitating permanent pacemaker therapy [2]. Therefore, the decision for catheter ablation of slow-pathway (CaSP) without prior ECG documentation of tachycardia should be made carefully.

In patients with clinical on–off tachycardia, in whom AVNRT can be induced during EPS, elimination or modulation of the SP is the first line therapy independent of pre-procedural electrocardiogram (ECG) documentation of the tachycardia [3, 4]. Furthermore, according to the current guidelines, empirical slow-pathway ablation can be considered in patients with clinical on–off tachycardia, in whom AVNRT cannot be induced during EPS, as long as there is proof of dual AV-nodal conduction properties and a maximum of one single echo-beat during EPS as well as pre-procedural ECG documentation of the tachycardia [3–6]. Several studies reported this empiric approach to be safe and feasible [7, 8].

However, clinical ECG documentation of paroxysmal on–off tachycardia is, despite all efforts, not infrequently unsuccessful. In these circumstances, the patients clinically suspected to have AVNRT are still referred to qualified centres for EPS without pre-procedural ECG documentation of the tachycardia. Based on current scientific evidence, it remains still unclear, if an empiric CaSP should be performed in patients without pre-procedural ECG documented tachycardia who have a dual AV-nodal conduction property and a maximum of one echo-beat.

Guidelines from 1995 recommended catheter ablation of SP in patients with clinically suspected AVNRT where there is evidence of dual AV-nodal conduction properties and a maximum of one echo-beat during EPS [5]. However, the current guidelines do not make a clear recommendation of CaSP for patients without pre-procedural ECG documentation of the tachycardia [3, 4]. Before a catheter ablation of SP in these patients, the benefit of possible elimination of covert AVNRT should be weighed against the risk of unnecessary AV-block despite of actual absence of AVNRT.

The aim of this study was to evaluate long-term results after CaSP in patients with non-ECG documented clinical on–off tachycardia in the presence of dual AV-nodal conduction properties and up to a single echo-beat.

## Methods

We retrospectively included patients who underwent catheter ablation of slow-pathway (CaSP) for clinical on–off tachycardia from the University Hospital Düsseldorf EPS registry from 2012 to 2015. In detail, the eligibility criteria are as follows; (1) patients with prior ECG documentation during tachycardia who had typical on–off tachycardia, or (2) patients with typical on–off palpitations with at least one successful termination using vagal manoeuvres (deep breathing/drinking cold water) but without prior ECG documentation who underwent at least two attempts at 24-h Holter monitoring.

### Electrophysiological study

All patients provided written informed consent prior to the EPS. All procedures were performed by two experienced operators with experience in more than 500 AVNRT ablations. The patients underwent mild sedation with midazolam (3–5 mg) and continuous propofol (5–10 mg/h). After placing three sheaths (6F, 8F, 8F) in the right or left femoral vein, three catheters (St. Jude Medical, Saint Paul, Minnesota, USA) were placed in the right ventricular apex (RV), at the His position and in the coronary sinus. Atrial and ventricular stimulation was performed with an external cardiac stimulator (UHS3000, Biotronik, Berlin, Germany). The intracardiac electrograms and electrocardiographic leads were displayed on a multichannel recording system (CardioLab, GE, USA) and recorded at a speed of 100 mm/s. Programmed stimulations from the RV and CS were conducted to induce AVNRT using one extrastimulus (S2), double extrastimuli (S3) and 3 extrastimuli (S4) with two different basic cycle lengths (CL) and atrial burst stimulation up to a minimum CL of 200 ms. If tachycardia was not inducible and no contraindications existed, metaproterenol (orciprenaline) was intravenously administered (0.25 mg, bolus) repeatedly until the heart rate increased by at least 20%, and programmed stimulations up to S4 with two different basic CL and atrial burst stimulation up to a minimum CL of 200 ms were conducted.

A jump phenomenon indicating dual AV-nodal physiology was defined as a prolongation of the AH-interval by more than 50 ms after a 10 ms decrease of the coupling interval during programmed extrastimulation. For mapping and CaSP we used a non-irrigated 4 mm catheter (AluCath Blu/Black, Biotronik, Berlin, Germany). The optimal ablation site was identified by electrophysiological signals and anatomically via fluoroscopy as previously described [9]. Radiofrequency energy was delivered when a maximal atrioventricular amplitude ratio of 0.5 was confirmed. Radiofrequency energy was titrated from 20 W up to 35 W and applied for 60 s if a steady junctional rhythm was confirmed during application. After a minimum of 20 min observation period, the same induction procedures were repeated to assess the endpoint of CaSP. The endpoint of CaSP was defined as the elimination of the slow-pathway (SP elimination) or a significant change of the SP conduction property with a jump phenomenon and up to a maximum of one echo-beat (SP modification). The elimination of echo-beat or the shortening of fast-pathway effective refractory period (ERP) greater than 20 ms was defined as SP modification. This ERP threshold (> 20 ms) was arbitrary defined based on the previous report of Lindsay et al. [9].

The patients were divided into two groups depending on the results of EPS. Patients with a dual AV-nodal conduction property and a maximum of one echo-beat but without pre-procedural ECG documentation of tachycardia, were assigned to the “pure empirical CaSP group” (Group-peCaSP). Patients with electrophysiologically inducible AVNRT as well as proof of two or more echo-beats with and without pre-procedural ECG documentation of tachycardia and patients with dual AV-nodal property and a maximum of one echo-beat with pre-procedural ECG documentation of tachycardia were assigned to the standard CaSP group (Group-stCaSP).

### Follow-up

Follow-up was conducted in our outpatient clinics, and by telephone communication with the patient as well as cooperation with the referring general practitioner involving with at least one 24-h Holter monitoring per year. Clinical outcome was assessed by absence/recurrence of clinical symptoms and/or ECG documentation.

New ECG documentation in patients with recurrence, persistence and/or occurrence of new symptoms was reviewed by two electrophysiologists. If they did not reach an agreement, an additional electrophysiologist reviewed the ECG and attempted to reach a consensus among the three.

### Study endpoints

The primary endpoint of the study was the documentation of other atrial tachyarrhythmias than AVNRT during follow-up. Secondary endpoints were complete elimination of clinical symptoms, recurrence of AVNRT, incidence of ablation induced AV block.

### Data analysis

Continuous data were shown as mean ± standard deviation (SD). Numerical data were shown in frequencies and proportions. Differences between groups were analysed by Chi-square test, fisher’s exact test or student’s *T* Test. Two-sided *P* value of < 0.05 was considered statistically significant. All authors have read and agreed to the manuscript as written.

## Results

### Patient population and EPS

**Fig. 1:**
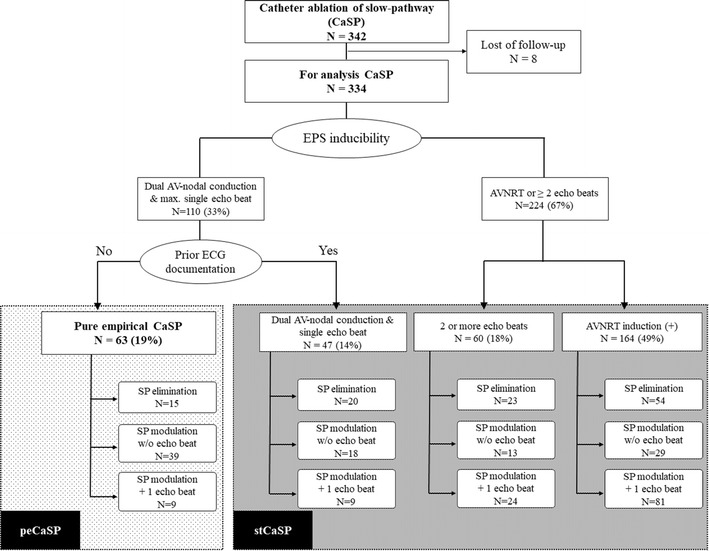
Schematic diagram of patient population and endpoints of catheter ablation. Out of 342 consecutive patients who underwent catheter ablation of slow-pathway (CaSP), 334 patients were routinely followed up after SPA. Pure empirical CaSP (see text) was performed in 63 patients without pre-procedural ECG documentation. AVNRT was induced in 164 patients and 2 or more echo-beats were noted in 60 patients

Our original patient population in the registry consisted of 341 patients who underwent CaSP due to paroxysms of palpitations and/or clinical ECG documentation. Eight patients were excluded (lost during follow-up) and consequently 334 patients (91 male, 47.5 ± 17.6 years) were included (Fig. 1).

Patient’ characteristics and the results of the EPS are shown in Table 1. Based on the results of EPS and prior ECG documentation, 63 patients were assigned to the Group-peCaSP. The remaining 271 patients were assigned to the Group-stCaSP. In 224 patients sustained AVNRT could be induced or the patients showed at least dual AVN properties with 2 or more AV-nodal echo-beats.

**Table 1:** Patient characteristics and results of electrophysiological study

	Total (*N* = 334)	peCaSP (*N* = 63)	stCaSP (*N* = 271)	*P* value (peCaS*P* vs stCaSP)
Male	91 (27%)	15 (24%)	76 (28%)	0.50
Age (years)	47.5 ± 17.6	41.4 ± 15.7	49.0 ± 17.8	0.0020
Basic EPS				
Dual AV-nodal conduction	314 (94%)	57 (90%)	257 (95%)	0.19
At least 1 echo-beat	288 (86%)	43 (68%)	245 (90%)	< 0.0001
2 or more echo-beats	191 (57%)	0 (0%)	191 (70%)	< 0.0001
AVNRT induction	146 (44%)	0 (0%)	146 (54%)	< 0.0001
Cycle length of AVNRT (ms)	386 ± 63	n.a.	386 ± 63	n.a.
Metaproterenol administration	145 (43%)	58 (92%)	87 (32%)	< 0.0001
Dual AV-nodal conduction	144/145 (99%)	57/58 (98%)	87/87 (100%)	0.40
Only after metaproterenol adm.	18/145 (12%)	5/58 (9%)	13/87 (15%)	0.26
At least 1 echo-beat	135/145 (93%)	50/58 (86%)	85/87 (98%)	0.015
Only after metaproterenol adm.	36/145 (25%)	12/58 (21%)	24/87 (28%)	0.35
2 or more echo-beats	44/145 (30%)	0/58 (0%)	44/87 (51%)	< 0.0001
Only after metaproterenol adm.	33/145 (23%)	0/58 (0%)	33/87 (38%)	< 0.0001
AVNRT induction	18/145 (12%)	0/58 (0%)	18/87 (21%)	0.0002
Cycle length of AVNRT (ms)	379 ± 74	n.a.	379 ± 74	n.a.

During EPS, AVNRT was induced without metaproterenol infusion in 146 patients (44%). In 191 patients (57%) dual AV-nodal conduction properties and 2 or more echo-beats were noted without metaproterenol infusion. In total, 145 patients (43%) received metaproterenol during EPS. Details of metaproterenol administration are also shown in Table 1. Two or more echo-beats were seen only after metaproterenol injection in 33 patients (23%).

### Catheter ablation

As the procedure endpoints (Fig. 1), SP elimination was noted in 112 cases (34%), and SP modification in the remaining 222 patients (66%). In these 222 patients with SP modulation, only AH-jump (99 patients, [45%]) and AH-jump with one echo-beat (123 patients, [55%]) were noted at the end of the procedure.

In Group-peCaSP (63 patients) at baseline EPS, AH-jump with one echo-beat was demonstrated in 55 patients and AH-jump without echo-beat was noted in 8 patients during baseline EPS. After CaSP, 9 patients out of these 63 showed AH-jump with one echo-beat. The endpoints of CaSP in this group were the elimination of echo-beat (46 patients), and the shortening of fast-pathway ERP (17 patients). Junctional rhythm was documented during radiofrequency application in all 63 patients.

All CaSP procedures were conducted without any major complications such as permanent AV block requiring pacemaker implantation, cardiac tamponade or arteriovenous fistula for which invasive interventions were necessary. Transient first-degree AV block was observed in 2 patients acutely after radiofrequency application (0.6%). At the end of the procedure, PQ intervals fully recovered to baseline values in all patients. A small arteriovenous fistula (shunt < 50 mL/min) and small groin hematoma, which were successfully treated by manual compression, were each noted in 1 patient. Hemodynamically irrelevant pericardial effusion under 3 mm was noted in 2 patients. These cases were clinically observed without any additional interventions.

### Follow-up

During the mean follow-up of 909 ± 435 days, other atrial tachyarrhythmias were documented in 6 patients (1.8%). The documented tachyarrhythmias were atrial flutter in one case, focal atrial tachycardia in two cases and inappropriate sinus tachycardia in three cases. In these 6 patients, clinical symptoms were similar to the symptoms described prior to EPS and the tachycardia was eventually documented with repeated Holter ECG monitoring. The patients with atrial flutter and focal atrial tachycardia underwent a second EPS with successful catheter ablation.

As for the clinical symptoms, 258 patients (77%) had no recurrence during the follow-up period. Patients with a pre-procedural ECG documentation of the tachycardia tended to be more likely to be symptom-free compared to patients without pre-procedural ECG documentation of the tachycardia (81% vs 73%, *P* = 0.057). There was no significant difference in the elimination of symptoms between patients with SP elimination and SP modulation (90/112 [80%] vs 168/222 [76%], *P* = 0.34).

Four patients (1.2%) developed recurrence of on–off tachycardia documented by ECG. They underwent a 2nd EPS and demonstrated AVNRT-induction (1 patient) and inducibility of two or more echo-beats (2 patients) during Re-do EPS. A maximum of 1 echo-beat with dual pathway conduction properties was noted in 1 patient. These patients showed the same inducibility in the 2nd EPS as was observed in the 1st EPS. They were free of symptoms after the second CaSP. There was no significant difference in the recurrence rate of AVNRT between patients with SP elimination and SP modulation (0/112 [0%] vs 4/222 [1.8%]. *P* = 0.31).

### Pure empirical SP ablation

**Fig. 2:**
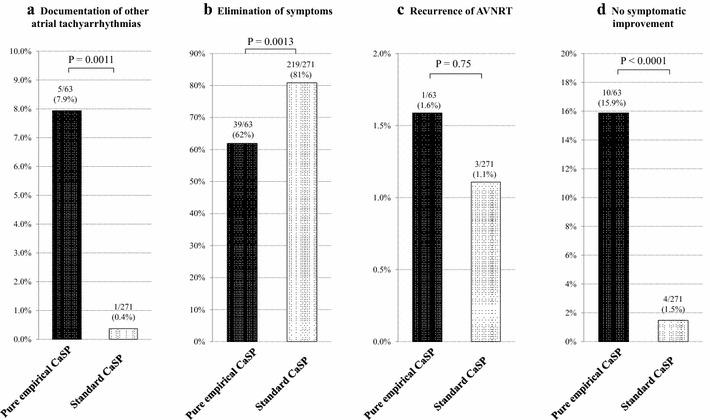
Pure Empirical Slow-Pathway Ablation and Follow-up Results. **a** Patients who underwent pure empirical catheter ablation of slow-pathway (CaSP) showed significantly higher incidence of other atrial tachycardia documentation during follow-up. **b** Patients who underwent pure empirical CaSP showed significantly lower complete symptom elimination during follow-up as compared to patients with standard CaSP. **c** Between patients with pure empirical CaSP and standard CaSP there was no significant difference in the recurrence of AVNRT. **d** There was significantly higher incidence of no symptomatic improvement in patients after pure empirical CaSP as compared to those after standard CaSP. *P* values were calculated with Fisher exact test (**a**, **c**, **d**) and with Chi-square test (**b**)

A significantly higher incidence of other atrial tachyarrhythmias documentation during follow-up was noted in the Group-peCaSP patients (Fig. 2a; 5/63 [7.9%] vs 1/27 [0.4%], *P* = 0.0011).

The patients with peCaSP showed a significantly lower percentage of clinical symptom elimination as compared to those with stCaSP (Fig. 2b; 39/63 [62%] vs 219/271 [81%], *P* = 0.0013). There was no significant difference in the rate of AVNRT recurrence in the two groups (Fig. 2c; 1/63 [1.6%] vs 3/271 [1.1%], *P* = 0.75).

Significantly more patients who underwent pure empirical CaSP complained of no symptomatic improvement compared to those with standard CaSP (Fig. 2d; 10/63 [15.9%] vs 4/271 [1.5%], *P* < 0.0001).

All 6 patients with minor complications (2 with transient first-degree AV block, 1 with arteriovenous fistula, 1 with groin hematoma, 2 with pericardial effusion) belonged to the Group-stCaSP, but the incidence of complication showed no significant difference between Group-peCaSP and Group-stCaSP (0/63 [0%] vs 6/271 [2.2%], *P* = 0.60).

## Discussion

The main findings of the present study are (1) catheter ablation of SP in on–off tachycardia patients who showed dual AV-nodal conduction property with up to 1 echo-beat and without pre-procedural ECG documentation (“pure empirical” CaSP), was associated with higher incidence of subsequent documentation of other atrial tachyarrhythmias, (2) pure empirical CaSP was also associated with higher persistence of clinical symptoms. On the other hand, over 60% of the patients after pure empirical CaSP can live without any symptom recurrence. To the best of our knowledge, this is a first detailed cohort study assessing the validity of “pure empirical” catheter ablation of slow-pathway approach.

The slow-pathway ablation is associated with low risk and high success rates. Therefore, CaSP has been recognized as a class I therapy in patients with diagnosed AVNRT [10]. However, in some cases with on–off tachycardia, it is difficult to induce the tachycardia or to prove the presence of AVNRT during EPS. In fact, sustained AVNRT was only induced in 164 patients (49%) in our study, including induction under metaproterenol infusion.

The current guideline supports empirical CaSP when AVNRT is not inducible but with tachycardia ECG documentation [6].

On the other hand, several authors described the empiric catheter ablation of SP without prior ECG documentation to be safe and feasible [7, 8]. A current analysis of 32 out of 3003 patients by Wegner et al. showed that slow-pathway modulation in patients with only two echo-beats was safe and effective [11]. Lin et al. reported effectiveness of slow-pathway modulation even in the presence of one or two echo-beats without inducibility of AVNRT but documented tachycardia [12]. Bogun et al. showed effectiveness of ablation in patients with documented tachycardia without inducibility but proof of dual AV nodal pathway or echo-beats [13]. These data, however, were obtained under the specified condition of ECG documented tachycardias.

It still remains unclear if CaSP should be performed in patients with supraventricular tachycardia without pre-procedural ECG documentation and with dual AV-nodal conduction property with single echo-beats. In our study, pure empirical CaSP provided complete subjective symptom suppression in over 60% of patients with on–off tachycardia without pre-procedural ECG documentation, despite all efforts to detect tachycardia with Holter monitoring. The patients in the peCaSP group had severe symptoms which significantly affected subjective quality-of-life and, therefore, were referred to our institute despite no previous ECG documentation. In this patient population with a strong preference for more effective treatment, only completing the EPS without ablation and continuing further ECG monitoring to document the tachycardia may be less than optimal. The present study provides a possible clue to improve their symptoms although our study is underpowered for safety assessment.

Meanwhile our data demonstrated a significantly higher incidence of other atrial tachyarrhythmias after the pure empirical CaSP as well as a higher persistence of clinical symptoms. Considering the possible risk of AV block after CaSP, mere pure empirical CaSP may be also suboptimal to fully live up to patient’ needs.

### Clinical implications

With technological development, many portable ECG monitoring devices have emerged. Therefore, the effort to document an ECG during tachycardia should be taken as far as possible, to prevent unnecessary SPA and consequently unnecessary complications such as AV-block. The discrepancy between the incidence of recurrent symptoms (78/334 patients, 23%) and the documented AVNRT recurrence (4/334 patients, 1.2%) in the present study underscores the importance of these devices, which will be able to clarify the pathology.

In all these circumstances, the patients with highly symptomatic tachycardias which cannot be documented even by current portable monitoring devices may be indicated for pure empirical CaSP. After pure empirical CaSP, careful monitoring should be conducted due to possible documentation of other atrial tachyarrhythmias.

### Limitations

There are some limitations in the present study. First, this is a single centre cohort study. Second, patients with other atrial tachyarrhythmia which was demonstrated during follow-up might have also had AVNRT at the time of CaSP. Due to the non-inducibility of AVNRT during EPS, this possibility could not be excluded. Third, metaproterenol, instead of isoproterenol was administered in the case of non-inducibility because of our institutional standard and no official approval for isoproterenol in Germany. Another group from Germany also utilized metaproterenol at the same dose for AVNRT induction [7]. Additionally, also due to our institutional standard, intravenous sedation was adopted in our study, which may affect the inducibility of tachycardia. However, our strategy can be validated based on the recurrence rate of AVNRT in the standard SPA patients with ECG documentation (under 2%) and the complication rate as low as that of the reference data [14]. Feldman et al. also reported previously that there were no differences in procedural outcomes under general anesthesia [15]. We could not exclude the placebo effect after pure empirical CaSP. In the present study, we had no major complications including permanent AV-block. However, our study is underpowered for the safety assessment due to the patient number. Finally, regarding the shortening of effective refractory period as an endpoint of CaSP, the autonomic tone variation cannot be excluded.

## Conclusions

Higher incidence of other AT and higher persistence of subjective symptoms are demonstrated after pure empirical slow-pathway ablation. However, pure empirical slow-pathway ablation improves clinical symptoms in 60% of patients with non-documented on–off tachycardia. Careful monitoring after pure empirical slow-pathway ablation should be conducted due to possible documentation of other atrial arrhythmias.
